# Retrospective Analysis of Fungal Isolations in Patients on Veno-Venous Extracorporeal Membrane Oxygenation: The Multicenter RANGER STUDY 2.0

**DOI:** 10.3390/jof11050377

**Published:** 2025-05-15

**Authors:** Annalisa Boscolo, Andrea Bruni, Marco Giani, Eugenio Garofalo, Nicolò Sella, Tommaso Pettenuzzo, Arianna Peralta, Michela Bombino, Matteo Palcani, Emanuele Rezoagli, Matteo Pozzi, Elena Falcioni, Eugenio Biamonte, Francesco Murgolo, Leonardo Gottin, Federico Longhini, Salvatore Grasso, Paolo Navalesi, Giuseppe Foti

**Affiliations:** 1 Department of Medicine (DIMED), University of Padua, 35122 Padua, Italy; annalisa.boscolobozza@unipd.it; 2 Institute of Anesthesia and Critical Care, Padua University Hospital, 35128 Padua, Italy; n.sella89@gmail.com (N.S.); tommaso.pettenuzzo@aopd.veneto.it (T.P.); arianna.peralta@aopd.veneto.it (A.P.); 3 Department of Medical and Surgical Sciences, Magna Graecia University, 88100 Catanzaro, Italy; andreabruni@unicz.it (A.B.); eugenio.garofalo@unicz.it (E.G.); egbiam@yahoo.it (E.B.); flonghini@unicz.it (F.L.); 4 School of Medicine and Surgery, University of Milano Bicocca, 20900 Monza, Italy; marco.giani@unimib.it (M.G.); m.palcani@campus.unimib.it (M.P.); emanuele.rezoagli@unimib.it (E.R.); matteo.pozzi1@unimib.it (M.P.); giuseppe.foti@unimib.it (G.F.); 5 Department of Emergency and Intensive Care, Fondazione IRCSS San Gerardo dei Tintori, 20900 Monza, Italy; michela.bombino@irccs-sangerardo.it; 6 Department of Surgery, Dentistry, Paediatrics and Gynaecology, University of Verona, 37129 Verona, Italy; elena.falcioni@aovr.veneto.it (E.F.); leonardo.gottin@univr.it (L.G.); 7 Cardiothoracic and Vascular Intensive Care Unit, Verona University Hospital, 37126 Verona, Italy; 8 Department of Precision and Regenerative Medicine and Ionian Area, School of Medicine, University of Bari “Aldo Moro”, 70121 Bari, Italy; francesco.murgolo@uniba.it (F.M.); salvatore.grasso@uniba.it (S.G.)

**Keywords:** fungi, extracorporeal membrane oxygenation, ICU, Intensive Care Unit, isolation, infection, colonization, ECMO, V-V ECMO, extracorporeal membrane oxygenation

## Abstract

**Background:** Veno-venous extracorporeal membrane oxygenation (V-V ECMO) represents a progressively adopted life-sustaining intervention worldwide, particularly in the management of acute respiratory distress syndrome. Nevertheless, data concerning the prognostic significance of fungal isolation in this setting remain unclear. This study aims (i) to assess the incidence of fungal infection and colonization in a homogeneous cohort of V-V ECMO patients, and (ii) to evaluate the association between fungal infection or colonization and 1-year mortality, with a focus on the impact of specific fungal species. **Methods:** All consecutive adults admitted to the Intensive Care Units of five Italian university-affiliated hospitals and requiring V-V ECMO were screened. Exclusion criteria were age < 18 years, pregnancy, veno-arterial or mixed ECMO-configuration, incomplete records and survival < 24 h after V-V ECMO placement. A standard protocol of microbiological surveillance was applied and the distinction between different fungal species were made through in vivo and vitro tests. Cox-proportional hazards models, Kaplan–Meier curves and linear logistic regressions were applied for investigating mortality. **Results:** Two-hundred and seventy-nine V-V ECMO patients (72% male) were enrolled. The overall fungal isolation was 41% (n. 114): 23% infections and 18% colonizations. The overall 1-year mortality, among fungal isolations, was 40%, with no different risk in case of fungal infection (26 out of 63, 41%) (aHR 0.85, 95% CI [0.53–1.37], *p*-value 0.505) and colonization (20 out of 51, 39%) (aHR 0.86, 95%CI [0.51–1.43], *p*-value 0.556), as compared to patients never detecting fungi (68 out of 165, 41%, *reference*). According to the isolated mycotic species, as compared to *Candida* sp. group (*reference*), the risk of death was greater when different fungal species (e.g., *Aspergillus* sp. and *Candida* sp.) were concomitantly isolated in the same patient (OR 1.17, 95%CI [1.12–11.07], *p*-value 0.031. **Conclusions:** In the overall population, 23% V-V ECMO patients recorded ‘late’ fungal infections and 18% fungal colonizations, with a similar risk of death as compared to patients never experiencing fungi during the V-V ECMO course. The detection of concomitant different fungal species was an independent risk factor for 1-year mortality.

## 1. Background

Veno-venous extracorporeal membrane oxygenation (V-V ECMO) is increasingly adopted worldwide as a life-sustaining therapy, particularly in patients with severe acute respiratory distress syndrome (ARDS) unresponsive to conventional mechanical ventilation [1,2,3,4]. By providing extracorporeal gas exchange, V-V ECMO can fully substitute the lungs’ function in oxygen delivery and carbon dioxide removal [1,5,6,7]. According to the Extracorporeal Life Support Organization (ELSO) registry, more than 56,000 adult respiratory ECMO cases have been reported, emphasizing its growing significance in critical care [1]. Despite its life-saving potential in cases of respiratory failure, V-V ECMO is associated with a heightened risk of infections, including invasive fungal infections (IFIs). This increased susceptibility is multifactorial, involving prolonged ICU stays, extensive use of broad-spectrum antimicrobials and immune dysregulation due to critical illness [1,2,3,4,5]. While the overall burden of IFIs in ICU populations is well recognized, data specifically focused on IFIs among V-V ECMO recipients remain limited.

Since the initial definitions proposed by the European Organization for Research and Treatment of Cancer/Invasive Fungal Infections Cooperative Group (EORTC) in 2002 [8], considerable efforts have been dedicated to understanding the clinical implications of fungal infections (FIs). The latest guidelines from the European Society for Clinical Microbiology and Infectious Diseases (ESCMID), published in 2017, emphasize a multifaceted diagnostic approach involving clinical evaluation, radiological imaging, and both serological and microbiological testing [9]. Nonetheless, the incidence and outcomes of FIs in patients undergoing V-V ECMO support remain poorly characterized.

IFIs are well established as major contributors to morbidity and mortality, especially among immunocompromised individuals. However, IFIs also affect non-neutropenic critically ill patients, usually admitted to the ICU, even in the absence of traditional risk factors for fungal infections [10,11,12,13,14]. The recently published CHARTER-IFI study, which assessed over 700 ICU patients, reported an incidence of 4.0 fungal infections per 1.000 ICU admissions. However, it did not analyze outcomes specifically in patients receiving extracorporeal support [15]. Similarly, the Italian AURORA study identified 105 IFI episodes among 5.561 ICU patients over an 18-month period, with infections predominantly caused by yeasts rather than filamentous fungi—corresponding to overall incidences of 16.5 and 2.3 cases per 1.000 admissions, respectively. The crude mortality rate was alarmingly high (42.8%), particularly in cases of mold infections (61.5%) [16]. Yet, neither of these studies addressed the incidence or prognostic impact of IFIs in the context of V-V ECMO.

Building on these gaps, we designed the present multicenter retrospective study with the primary aim of investigating, in a large cohort of V-V ECMO patients, (i) the prevalence of fungal infections and colonization, and (ii) the associated 1-year mortality risk, compared to patients who did not experience fungal isolation during their V-V ECMO course, with an additional analysis based on fungal species.

## 2. Methods

This multicenter observational study was conducted between 1 January 2017 and 31 December 2022 in 5 ICU of Italian university-affiliated hospitals, overall accounting for a total of 70-ICU beds (i.e., Mater Domini Hospital (Catanzaro); Padua University Hospital; Verona University Hospital; Policlinico University Hospital (Bari) and Fondazione IRCSS Gerardo Hospital dei Tintori Hospital (Monza)). We included adult patients, over 18 years of age, who received V-V ECMO for respiratory support during the study period. The exclusion criteria were age < 18 years old, pregnancy, veno-arterial (V-A) or mixed ECMO-configuration (e.g., V-VA), incomplete records for the main outcomes (absence of 1-year mortality and/or microbiological surveillance) and survival <24 h after cannulation. The study was conducted in compliance with the Declaration of Helsinki and the approval for the investigation was granted by the local Ethics Committee “Comitato Etico Territoriale Regione Calabria” (approval n. 22 on 27 September 2023), which waived the need for informed consent due to the retrospective observational nature of the study. All patient data was anonymized and de-identified before analysis. This study followed the ‘Strengthening the Reporting of Observational Studies in Epidemiology (STROBE) statement guidelines for observational cohort studies’ (Supplementary Table S1) [17].

The decision to start V-V ECMO treatment was made by senior intensivists (PN, FL, EB, GF, LG, SG), according to the ELSO guidelines/recommendations [1]. All V-V ECMOs were placed exclusively in the ICU, and a femoro-jugular configuration was preferred. All centers kept the ECMO circuit isolated as much as possible (e.g., withdrawals or infused medications were not recommended). Antimicrobial prophylaxis, at the time of cannulation, was uniformly not administered [18]. Routine microbiological surveillance was uniformly conducted in all centers, starting from ICU admission, as previously published [18]. All positive bacterial and fungal cultures were independently evaluated, considering the available clinical, laboratory and radiographic data, by specialized intensivists and infectious diseases specialists. In case of fungal cultures, strict communication between ICUs, microbiology laboratories and infectious disease specialist consultants were mandatory before starting a targeted antifungal therapy. The microbiological follow-up for this study arbitrarily ended 72 h after V-V ECMO decannulation.

According to the FUNDICU consensus definition, published in 2024, V-V ECMO patients were distinguished in 3 groups: the ‘fungal infection’ group (i), including patients with the presence of fungal cultures, collected from bronchoalveolar lavage (BAL), soft tissue, blood stream, urine etc., and in the presence of symptoms, radiological and endobronchial changes, or the presence of histological changes consistent with fungal invasion of tissue [19,20,21]; the ‘fungal colonization’ group (ii), when mycological evidence was not supported by clinical symptoms, radiological or endobronchial changes; and the ‘absence of fungi’ group (iii), including the population without evidence of FIs or colonizations. Moreover, we also recorded some fungal biomarkers (i.e., galactomannan (GM) antigen and β-D-glucan), quantified concomitantly to microbiological cultures. Specifically, they were requested at least once in each patient with fungal infections, while the percentage was lower (around 60%) in the case of the colonization solely.

## 3. Data Collection

The electronic health records were retrospectively examined, and the following variables were collected: demographic and baseline characteristics, Charlson’s Comorbidity index, Sequential Organ Failure Assessment (SOFA) score at ICU admission and at V-V ECMO cannulation, initiation of invasive mechanical ventilation (IMV), indications for V-V ECMO support, year of V-V ECMO connection, overall V-V ECMO duration, concomitant isolation of viral or bacterial isolations (Table 1), outcomes of interest (1-year mortality, 28-day ventilator-free days, Intensive Care Unit (ICU) length of stay (LOS) [22], microbiological results obtained during V-V ECMO support, type of fungal isolation, site of isolation and fungal biomarkers [18] (Table 2).

## 4. Statistical Analysis

Continuous variables are presented as medians and interquartile ranges (IQR) or as mean and standard deviation (SD), while categorical variables are presented as numbers (percentages). Baseline patients’ characteristics and outcome variables were compared between two or three pre-defined subpopulations, as follows: (1) ‘fungal infections’, (2) ‘fungal colonitazions’, and (3) ‘absence of fungi’. The sample size could not be calculated due to the explorative design of our investigation and the scarcity of data on this topic. The *t*-test, Mann–Whitney test, ANOVA or Kruskal–Wallis test was properly used to compare continuous variables and adjusted by Benjamini–Hochberg method. Chi-square and Fisher’s exact tests were used for comparing categorical variables.

**Figure 1:**
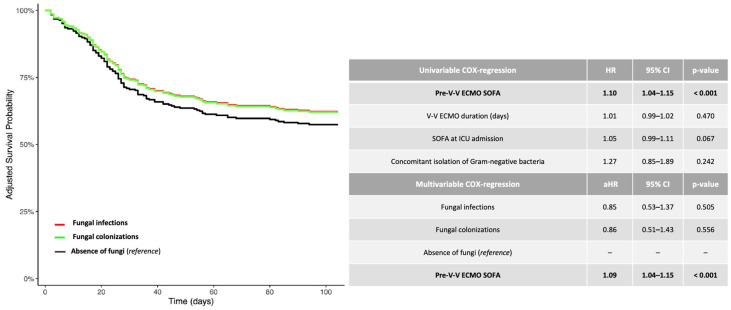
Adjusted 1-year survival curves (Cox regression model: fungal infection versus fungal colonization versus absence of fungi). The adjusted (covariate: SOFA score at V-V ECMO connection) HRs were calculated according to the univariable and multivariable Cox-proportional hazards models described in the table. Data are presented as HR, aHR and 95% CI. For unadjusted Kaplan–Meier survival curves, see Figure S2. Abbreviations: ECMO: extracorporeal membrane oxygenation; HR: hazard ratio; aHR: adjusted hazard ratio; CI: confidential interval; V-V: veno-venous.

**Figure 2:**
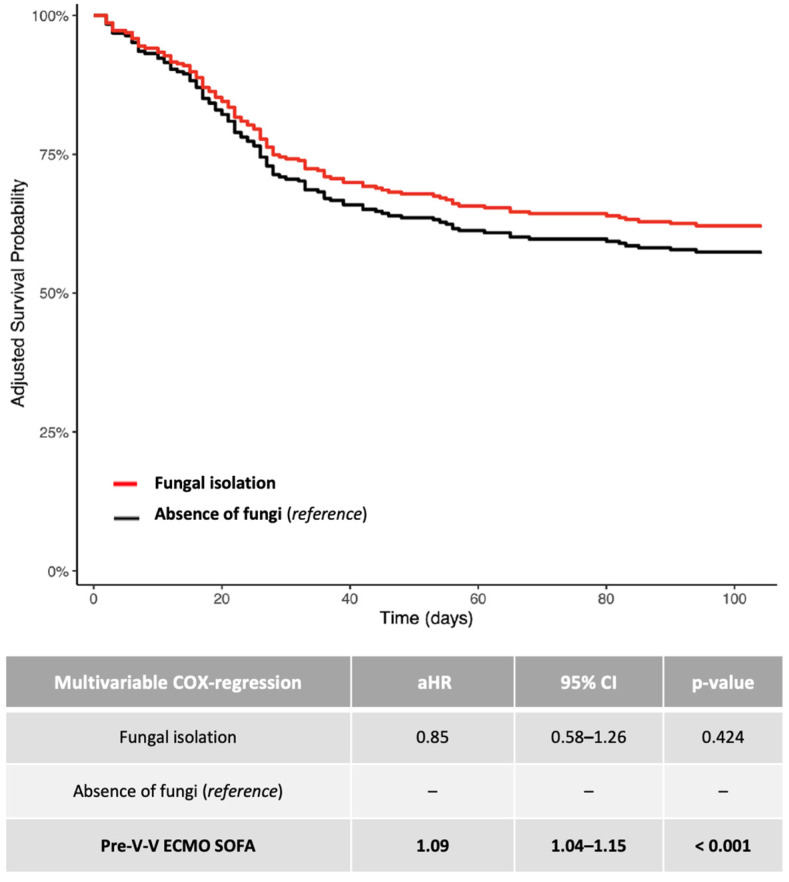
Adjusted 1-year survival curves (Cox regression model: fungal isolation versus absence of fungi). The adjusted (covariate: SOFA score at V-V ECMO connection) HRs were calculated according to the univariable and multivariable Cox-proportional hazards models described in the table. Data are presented as HR, aHR and 95% CI. For unadjusted Kaplan–Meier survival curves, see Figure S2. Abbreviations: ECMO: extracorporeal membrane oxygenation; HR: hazard ratio; aHR: adjusted hazard ratio; CI: confidential interval; V-V: veno-venous.

Regarding 1-year mortality, the Kaplan–Meier curves were provided only as graphical support (Figures S1 and S2). For investigating the risk of mortality, the adjusted hazard ratio (aHR) and 95% confidence intervals [CI] were calculated using Cox-proportional hazards models (Figure 1 and Figure 2). Cox-proportional hazards models assume that the hazard ratio is constant over time; therefore, the test for proportional hazard assumption was verified for each covariate included in the univariable model. The time-dependent variable started from ICU admission. All variables described in Table 1, with a significance *p*-value < 0.10, were included in an univariable Cox-proportional hazards model investigating 1-year mortality (*). Then, as shown in Figure 1 and Figure 2, the multivariable adjustment was provided according to significant confounders (*p*-value < 0.05) identified through the univariable Cox-proportional hazards model, as mentioned above (*). Given the low incidence of pre-existing fungal infections (~4%), a statistical analysis solely dedicated to this subgroup was not feasible. For investigating the impact of fungal isolation (in terms of species and site) on the risk of 1-year mortality, a linear logistic regression was applied (Table 3). The unadjusted odds ratio (OR) and 95% CI were calculated, all tests were two-sided and *p*-values < 0.05 were considered statistically significant. The analyses were performed using R (version 4.0.3, R foundation for Statistical Computing, Vienna, Austria).

## 5. Results

From January 2017 to December 2022, 482 ICU patients treated with ECMOs for severe respiratory failure were screened. After excluding 199 subjects needing V-A or mixed ECMO-configuration, 2 patients because of incomplete records and 2 due to a survival shorter than 24 h after V-V ECMO initiation, 279 patients (median age 54 years; 72% male) were included in the final analysis (see Figure S3). Patients’ demographic characteristics, SOFA scores, indications for V-V ECMO support, corticosteroids use and other baseline information are summarized in Table 1.

### 5.1. Baseline Characteristics and Primary Outcome

In our cohort, the prevalence of mycotic isolation among V-V ECMO patients was 42% (n. 114): among 279 patients, 63 (23%) were defined “infected” and 51 (18%) as “colonized”, while 165 (59%) subjects had no occurrence of fungi during extracorporeal treatment. Of those patients with fungal isolations, only 4 (4%) subjects had pre-detected fungal infections at the start of V-V ECMO, mostly *Aspergillus* sp. (time of the first fungal isolation: 2 [0–4] days after ICU admission). As described in Table 1, patients’ baseline characteristics were not different among the three subgroups, except for SOFA at the V-V ECMO connection that was significantly higher in case of fungal colonizations (9 [5–12]) as compared to other subgroups (*p*-value 0.008) (Table 1). As compared to patients never experiencing fungi, V-V ECMO duration was longer in the case of fungal isolation (infection: 16 [10–27] days, colonization: 16 [11–26] days) (*p*-value < 0.001); also, the length of ICU was remarkably more prolonged both in the case of fungal infection (35 [23–53] days) and colonization (35 [23–54]) days) (*p*-value < 0.001). Moreover, 49 out of 63 (78%) patients with fungal infection and 38 out of 51 (18%) patients with fungal colonization had a greater rate of concomitant GN bacteria isolation, usually detected before fungal isolation (*p*-value 0.007) (Table 1).

**Table 1:** Patients’ characteristics and outcomes.

	Overall Population (N = 279, 100%)	Fungal Infections ^1^ (N = 63, 23%)	Fungal Colonizations ^2^ (N = 51, 18%)	Others ^3^ (N = 165, 59%)	*p*-Value
**Baseline characteristics**					
Age, years	54 [44–61]	54 [45–59]	51 [38–63]	55 [45–61]	0.636
Gender (male), n (%)	200 (72)	44 (70)	36 (71)	120 (72)	0.146
TBW, Kg	66 [60–71]	67 [58–71]	67 [61–72]	65 [57–70]	0.583
Charlson Comorbidity Index (w/o age)	1 [0–2]	1 [0–2]	1 [0–1]	1 [0–2]	0.610
Sepsis Organ Failure Assessment at ICU admission	8 [6–11]	8 [6–9]	8 [4–10]	10 [7–12]	0.065 *
at V-V ECMO connection	9 [7–12]	8 [7–9]	9 [5–12]	9 [7–12]	**0.008** ^a^
Mechanical ventilation before V-V ECMO connection, days	2 [1–5]	3 [1–5]	2 [1–6]	2 [1–5]	0.113
Corticosteroids > 7 days	271 (97)	60 (95)	51 (100)	160 (97)	0.999
Indications for V-V ECMO support					
Acute respiratory distress syndrome, n (%)	233 (84)	61 (97)	37 (73)	135 (82)	0.767
Trauma, major burn, autoimmune disease, CLAD, n (%)	46 (16)	15 (24)	11 (22)	30 (18)	0.767
Year of V-V ECMO connection, n (%)					
2017–2019	101 (36)	18 (29)	22 (43)	61 (40)	0.266
2020–2022	178 (64)	45 (71)	29 (57)	104 (63)	0.266
V-V ECMO duration, days	12 [8–22]	16 [10–27]	16 [11–26]	11 [7–17]	<0.001 ^b^*
**Concomitant isolation of**					
Sars-CoV-2, influenza virus, n (%)	157 (56)	28 (44)	31 (61)	98 (59)	0.103
Gram-negative bacteria ^c^, n (%)	183 (66)	49 (78)	38 (75)	96 (58)	**0.007 ^e^**
MDR/ESBL profile	139 (50)	34 (54) ^d^	27 (53) ^d^	78 (47)	0.200
Multisensitive profile	44 (16)	15 (24)	11 (22)	18 (11)	0.200
Gram-positive bacteria, n (%)	57 (20)	12 (19)	14 (27)	31 (19)	0.393
**Outcomes**					
Mortality at 1-year, n (%)	116 (42)	26 (41)	20 (39)	68 (41)	0.970
28-day ventilator-free days	0 [0–8]	0 [0–5]	0 [0–6]	0 [0–9]	0.478
ICU LOS, days	27 [18–43]	35 [23–53]	35 [23–54]	23 [16–34]	**<0.001 ^f^**

Data are presented as absolute frequency (% of the included patients) or as median and interquartile range or as mean ± SD. Three groups are compared and named ‘1’, ‘2’ and ‘3’. ^a^: (1) vs. (2) *p*-value 0.039; ^b^: (1) vs. (3) *p*-value < 0.001, (2) vs. (3) *p*-value < 0.001; ^c^: in the case of multiple bacterial isolations, only the worst resistance pattern was counted. ^d^: 17 MDR/ESBL Gram-negative bacteria were detected before fungal infections, while 6 were detected before fungal colonizations (*p*-value 0.028); ^e^: (1) vs. (3) *p*-value 0.006, (2) vs. (3) *p*-value 0.047; ^f^: (1) vs. (3) *p*-value < 0.001, (2) vs. (3) *p*-value < 0.001. *: covariates available for univariable analysis. Abbreviations: ICU: intensive Care Unit; IBW: ideal body weight; ECMO: extracorporeal membrane oxygenation; N or n: number; SD: standard deviation; V-V: veno-venous; CLAD: chronic lung allograft dysfunction; ESBL: extended spectrum beta-lactamases; MDR: multidrug resistant.

### 5.2. Secondary Outcomes

As shown in Table 2, *Aspergillus* sp. (40%) was more frequently detected in case of infection, as compared to colonization (33%), while *Candida* sp. was prevalent in case of mycotic colonization (63% versus 38%) (overall *p*-value 0.004). 

**Table 2:** Microbiological characteristics.

	Fungal Infections (N = 63, 100%)	Fungal Colonizations (N = 51, 100%)	*p*-Value	
**Microbiological pattern**			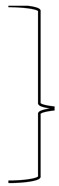	**0.004**
*Candida* sp ^a^., n (%)	24 (38)	32 (63)		
*Aspergillus* sp ^b^., n (%)	25 (40)	17 (33)		
Concomitant different *Fungal* sp ^c^., n (%)	14 (22)	2 (4)		
**Site of fungal isolation**				
airways, n (%)	24 (38)	45 (88)	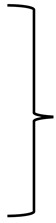	**<0.001**
blood stream, n (%)	11 (17)	0 (0)		
airway and blood stream, n (%)	9 (14)	0 (0)		
urinary tract, n (%)	15 (24)	1 (2)		
others (i.e., soft tissue etc.), n (%)	4 (6)	5 (10)		
**Fungal biomarkers**				
BAL galactomannan antigen > 1 ratio, n (%)	18 (29)	2 (4)	**<0.001**	
β-D-glucan (reference > 80 pg/mL), n (%)	8 (13)	3 (6)	0.340	

Data are presented as absolute frequency (% of the included patients). For more details about survival, see Figure 1 and Figure 2. ^a^: time of isolation 8 [5,6,7,8,9,10,11,12,13,14] days after ICU admission; ^b^: time of isolation 11 [7,8,9,10,11,12,13,14,15,16] days after ICU admission, except in pre-detected patients; ^c^: Different fungal isolations were included in this group. Abbreviations: N or n: number; sp.: species; BAL, bronchoalveolar lavage; ICU, Intensive Care Unit.

With regards to infections, many fungi were isolated from airways (38%) (i.e., mostly *Aspergillus* sp.) or from urinary tract (24%) (i.e., mainly *Candida* sp.); while, considering fungal colonizations, mycotic isolations from airways were prevalent (88%) (overall *p*-value < 0.001). BAL galactomannan antigen was usually greater than 1 in case of infection, as compared to colonization (overall *p*-value < 0.001), while no differences were found considering β-D-glucan (reference > 80 pg/mL) (*p*-value 0.340).

As shown in Table 1, the 1-year mortality was not different in patients experiencing fungal infection (26 out of 63, 41%) (aHR 0.85, 95% CI [0.53–1.37], *p*-value 0.505) or fungal colonization (20 out of 51, 39%) (aHR 0.86, 95% CI [0.51–1.43], *p*-value 0.556), as compared to those patients without mycotic isolation (68 out of 165, 41%, *reference*) (Figure 1).

Additional summary data on patients with documented fungal isolation are reported in Figure 2.

According to the type of isolated mycotic species, as compared to those patients culturing *Candida* sp. solely (*reference*), the risk of death was significantly greater in patients detecting simultaneously different fungal species (OR 1.17, 95% CI [1.12–11.07], *p*-value 0.031), while not in the case of *Aspergillus* sp. solely (OR 1.04, 95% CI [0.31–3.50], *p*-value 0.952), (Table 3). Finally, the site of isolation was not correlated to death (Table 3).

**Table 3:** Unadjusted odds of 1-year mortality according to isolated fungal species and site of detection.

Linear Logistic Regression	aOR	95% CI	*p*-Value
*Aspergillus* sp.	1.04	0.31–3.50	0.952
**Concomitant different *fungal* sp.**	**1.17**	**1.12–11.07**	**0.031**
*Candida* sp. (*reference*)	**-**	**-**	***-***
Isolation from airways and/or blood stream (*reference*: other sites)	1.17	0.27–5.06	0.831

*Abbreviations*: OR: odds ratio; CI: confidential interval; sp., species.

## 6. Discussion

In this multicenter retrospective study, we found that among 279 consecutive adult patients supported by V-V ECMO for acute respiratory failure, the prevalence of fungal colonization was 18%, while the prevalence of mycotic infection was 23% during V-V ECMO course. The 1-year mortality was not different in patients experiencing fungal infections (41%) or fungal colonizations (39%), as compared to those patients without mycotic isolation (41%), while the risk of death was greater in patients where different species of fungi were concomitantly isolated, as compared to one species.

In the past decade, IFIs have emerged as a growing concern in the ICU setting and recognized as a cause of morbidity and mortality not only in immunocompromised patients but also in the general critically ill population [22]. Recent studies have highlighted a rising incidence of IFIs among ICU patients, with estimates indicating that up to 20% may develop fungal infections during hospitalization [16,23].

The COVID-19 pandemic further altered the epidemiological landscape of fungal infections in critically ill patients [15]. As the pandemic progressed, clinicians increasingly focused on identifying and managing co-infections, particularly invasive pulmonary aspergillosis (IPA) [24]. This heightened awareness contributed to improved recognition and reporting of fungal infections in ICU settings [21,25]. For instance, mortality rates in COVID-19-Associated Pulmonary Aspergillosis have been reported to range from 30% to over 50%, especially when antifungal treatment is delayed—highlighting the need for heightened clinical vigilance, standardized diagnostic criteria, and early therapeutic interventions [26,27,28].

Corroborating this alarming trend, our study found fungal isolation in nearly half of the patients. This rate appears notably higher than those previously reported in studies of mixed ECMO populations [29], as well as in both V-A [30] and V-V ECMO cohorts [31]. This discrepancy may be explained by the fact that these earlier studies were more focused on proven infections rather than on fungal isolations. Moreover, both abovementioned investigations did not include COVID-19 patients, as we did.

A systematic review confirmed that critically ill patients on ECMO are more likely to develop candidemia and other fungal infections compared to those not receiving ECMO support [32]. Several risk factors contribute to this elevated vulnerability. Many ECMO patients have pre-existing comorbidities such as diabetes, cancer, or chronic lung disease, which predispose them to IFIs [30,33]. Additionally, the acute illness often leads to malnutrition or altered nutritional status, further weakening immune defenses [33].

With regards to the fact that in our cohort, the isolation of fungi was not necessarily related to a poorer outcome, we can speculate that fungal infections acquired during V-V ECMO support tend to occur relatively late. These infections are likely not severe enough to impact late mortality among patients who survive longer. This observation may also explain why, in the group without fungal isolations—which experienced a higher rate of early mortality, moslty due to bacterial or viral infections—fewer patients survived long enough to be at risk of acquiring a fungal infection. Therefore, the acquisition of such infections does not appear to be a clinically relevant factor influencing prognosis. Indeed, the *p*-values comparing the black and red curves (Figure 1 and Figure 2) were not statistically significant.

This interpretation is consistent with the previous literature reporting that fungal infections during ECMO typically develop later in the course of treatment and are more often associated with prolonged support or underlying risk factors, rather than being direct contributors to early mortality [34,35].

Moreover, broad-spectrum antibiotics, frequently administered to these V-V ECMO patients to combat pre-detected bacterial infections, can disrupt the microbiome and promote fungal overgrowth [18,36]. We attempted to explore this potential association indirectly by including the variable ‘concomitant isolation of GN bacteria’ in the multivariable Cox regression analysis, as shown in Figure 1. Unfortunately, this variable did not reach statistical significance in our cohort. However, our findings should be confirmed by larger prospective studies. Additionally, immunosuppressive therapies—including corticosteroids—further exacerbate the risk of Fis [36,37].

Moreover, the extensive use of invasive devices, such as central venous catheters, endotracheal tubes, and arterial lines, which are common in ECMO-supported patients, increases the risk of fungal colonization and subsequent infection [33,36]. Prolonged ICU stays, often necessary for ECMO, further increase exposure to these risks [33,36].

With regards to mortality, in our cohort, the overall survival rate aligns with data from the ELSO registry, which reports a 48% survival to hospital discharge in patients undergoing either V-A or V-V ECMO [29]. However, the current literature lacks specific data on the prognostic impact of fungal isolation—including both colonization and invasive infection—in patients receiving V-V ECMO. Nonetheless, the clinical relevance of IFIs as drivers of morbidity and mortality in ICU patients, particularly among immunocompromised individuals, is well documented [21]. For example, the Italian AURORA study reported 105 IFI episodes among 5.561 ICU patients over 18 months, with a crude mortality exceeding 40%, and reaching over 60% in cases of mold infections [16]. In particular, invasive aspergillosis is associated with significantly higher mortality than candidemia in critically ill or immunocompromised patients, with rates often exceeding 50% compared to 20–40% for invasive candidiasis [28,38,39,40]. However, in our V-V ECMO cohort, only the concomitant isolation of different mycotic species, usually including *Aspergillus* sp. and *Candida* sp., was linked to a higher risk of 1-year mortality.

Indeed, in the high-risk setting of V-V ECMO, where fungal isolation is strikingly common, early recognition and prompt intervention are paramount—not only to curb the progression from colonization to invasive infection, but potentially to alter the trajectory of patient survival.

This study had several limitations. First, it is a retrospective observational study which bears the limits of this design. Second, despite a wide population consisting exclusively of V-V ECMO adult patients, the categorization of the cohort according to the fungal isolation status inevitably resulted in three small-size subgroups. We believe that a broader cohort in the future would better delineate outcomes and validate our findings. Third, we did not investigate whether the cannulation site may influence the infectious risk, despite the internal jugular and femoral vein being the most common sites of catheterization (>80% in our cohort) [41]. Fourth, according to our previous findings, describing an inverse relationship between MDR GN bacteria occurrence and local institutional experience, we cannot exclude also a higher rate of fungal infection occurring in those centers with a lower annual hospital V-V ECMO volume [18].

## 7. Conclusions

In the overall population, 23% V-V ECMO patients recorded fungal infections and 18% recorded mycotic colonizations (of those, 40% died within 1 year), with a similar risk of death as compared to patients never experiencing fungi during V-V ECMO course. The detection of concomitant different fungal species was an independent risk factor for 1-year mortality.

## Data Availability

The datasets used and/or analyzed during the current study are available from the corresponding author on reasonable request.

## Supplementary Materials

The following supporting information can be downloaded at: https://www.mdpi.com/article/10.3390/jof11050377/s1; Table S1. STROBE Statement—Checklist; Figure S1. 1 year-mortality (Kaplan Meier: fungal isolation versus absence of fungi); Figure S2. 1 year-mortality; Figure S3. Flowchart; (Kaplan Meier: fungal infection versus fungal colonization versus absence of fungi)

## List of Abbreviations

ARDS	Acute respiratory distress syndrome
BAL	Bronchoalveolar lavage
CI	Confidence interval
COVID-19	Coronavirus disease 2019
ECMO	Extracorporeal membrane oxygenation
ELSO	Extracorporeal Life Support Organization
ESCMID	European Society for Clinical Microbiology and Infectious Diseases
EORTC	European Organization for Research and Treatment of Cancer
FIs	Fungal infections
GN	Gram-negative
GM	Galactomannan
ICU	Intensive Care Unit
IFI(s)	Invasive fungal infection(s)
IMV	Invasive mechanical ventilation
IQR	Interquartile range
LOS	Length of stay
MDR	Multi-drug resistant
OR	Odds ratio
PCR	Polymerase chain reaction
SD	Standard deviation
SOFA	Sequential Organ Failure Assessment
STROBE	Strengthening the Reporting of Observational Studies in Epidemiology
V-A ECMO	Veno-arterial extracorporeal membrane oxygenation
V-V ECMO	Veno-venous extracorporeal membrane oxygenation
V-VA ECMO	Veno-veno-arterial extracorporeal membrane oxygenation
