# Supplementary material for: Retrospective Analysis of Fungal Isolations in Patients on Veno-Venous Extracorporeal Membrane Oxygenation: The Multicenter RANGER STUDY 2.0
Source: J Fungi (Basel). 2025 May 15;11(5):377. doi: 10.3390/jof11050377 (PMC12113069; doi:10.3390/jof11050377)
Supplement: Supplementary file 1 [file jof-11-00377-s001.zip › jof-3620991-supplementary.pdf]

ADDITIONAL FILES

ARTICLE TITLE: Retrospective ANalysis of fUnGal infEctions in patients on veno-venous extracorporeal membrane oxygenation: The multicenter RANGER STUDY 2.0

Table S1. STROBE Statement—Checklist[1]

Figure S1. Flowchart

Figure S2. 1 year-mortality (Kaplan Meier: fungal isolation versus absence of fungi)

Figure S3. 1 year-mortality (Kaplan Meier: fungal infection versus fungal colonization versus absence of fungi)

REFERENCES

Additional-Table S1. STROBE Statement—Checklist[1]

	Item No	Recommendation	Page No
**Title and abstract**	1	(*a*) Indicate the study's design with a commonly used term in the title or the abstract	1, 4-5
		(*b*) Provide in the abstract an informative and balanced summary of what was done and what was found	
**Introduction**			
Background/rationale	2	Explain the scientific background and rationale for the investigation being reported	5
Objectives	3	State specific objectives, including any prespecified hypotheses	6
**Methods**			
Study design	4	Present key elements of study design early in the paper	7, 8
Setting	5	Describe the setting, locations, and relevant dates, including periods of recruitment, exposure, follow-up, and data collection	7, 8
Participants	6	(*a*) Give the eligibility criteria, and the sources and methods of selection of participants. Describe methods of follow-up	7
		(*b*) For matched studies, give matching criteria and number of exposed and unexposed	--
Variables	7	Clearly define all outcomes, exposures, predictors, potential confounders, and effect modifiers. Give diagnostic criteria, if applicable	8, 9
Data sources/ measurement	8*	For each variable of interest, give sources of data and details of methods of assessment (measurement). Describe comparability of assessment methods if there is more than one group	10

Bias	9	Describe any efforts to address potential sources of bias	-
Study size	10	Explain how the study size was arrived at	10
Quantitative variables	11	Explain how quantitative variables were handled in the analyses. If applicable, describe which groupings were chosen and why	10, 11
Statistical methods	12	(*a*) Describe all statistical methods, including those used to control for confounding	10, 11
		(*b*) Describe any methods used to examine subgroups and interactions	
		(*c*) Explain how missing data were addressed	
		(*d*) If applicable, explain how loss to follow-up was addressed	-
		(*e*) Describe any sensitivity analyses	-
**Results**			
Participants	13*	(a) Report numbers of individuals at each stage of study—eg numbers potentially eligible, examined for eligibility, confirmed eligible, included in the study, completing follow-up, and analysed	11
		(b) Give reasons for non-participation at each stage	
		(c) Consider use of a flow diagram	
Descriptive data	14*	(a) Give characteristics of study participants (eg demographic, clinical, social) and information on exposures and potential confounders	11-12
		(b) Indicate number of participants with missing data for each variable of interest	-
		(c) Summarise follow-up time (eg, average and total amount)	-
Outcome data	15*	Report numbers of outcome events or summary measures over time	11, 12
Main results	16	(a) Give unadjusted estimates and, if applicable, confounder-adjusted estimates and their precision (eg, 95% confidence interval). Make clear which confounders were adjusted for and why they were included<br>(b) Report category boundaries when continuous variables were categorized	11-13

		(c) If relevant, consider translating estimates of relative risk into absolute risk for a meaningful time period	
Other analyses	17	Report other analyses done—eg analyses of subgroups and interactions, and sensitivity analyses	-
**Discussion**			
Key results	18	Summarise key results with reference to study objectives	13-15
Limitations	19	Discuss limitations of the study, taking into account sources of potential bias or imprecision.<br><br>Discuss both direction and magnitude of any potential bias	16
Interpretation	20	Give a cautious overall interpretation of results considering objectives, limitations, multiplicity of analyses, results from similar studies, and other relevant evidence	17
Generalisability	21	Discuss the generalisability (external validity) of the study results	-
**Other information**			
Funding	22	Give the source of funding and the role of the funders for the present study and, if applicable, for the original study on which the present article is based	20, 21

Additional-Figure S1. 1 year-mortality (Kaplan Meyer: fungal isolation versus absence of fungi)

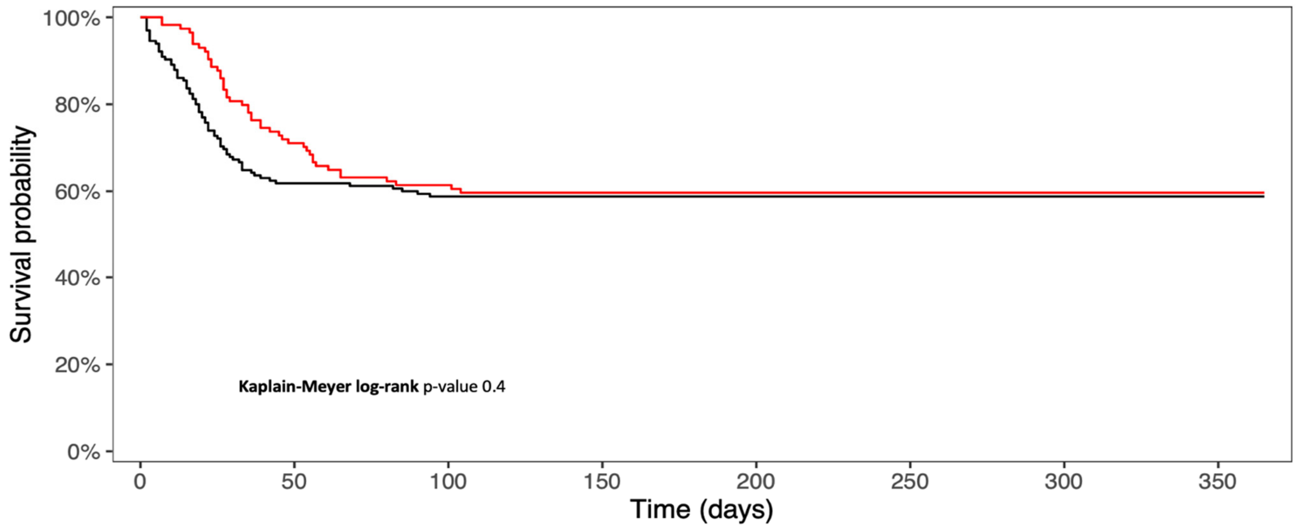

At Risk

Absence of fungi	165	102	97	97	97	97	97	97
Fungal infections	114	81	70	68	68	68	68	68

Events

Absence of fungi (black)	0	63	68	68	68	68	68	68
Fungal infections (red)	0	33	44	46	46	46	46	46

Additional-Figure S2. 1 year-mortality (Kaplan Meyer: fungal infection versus fungal colonization versus absense of fungi)

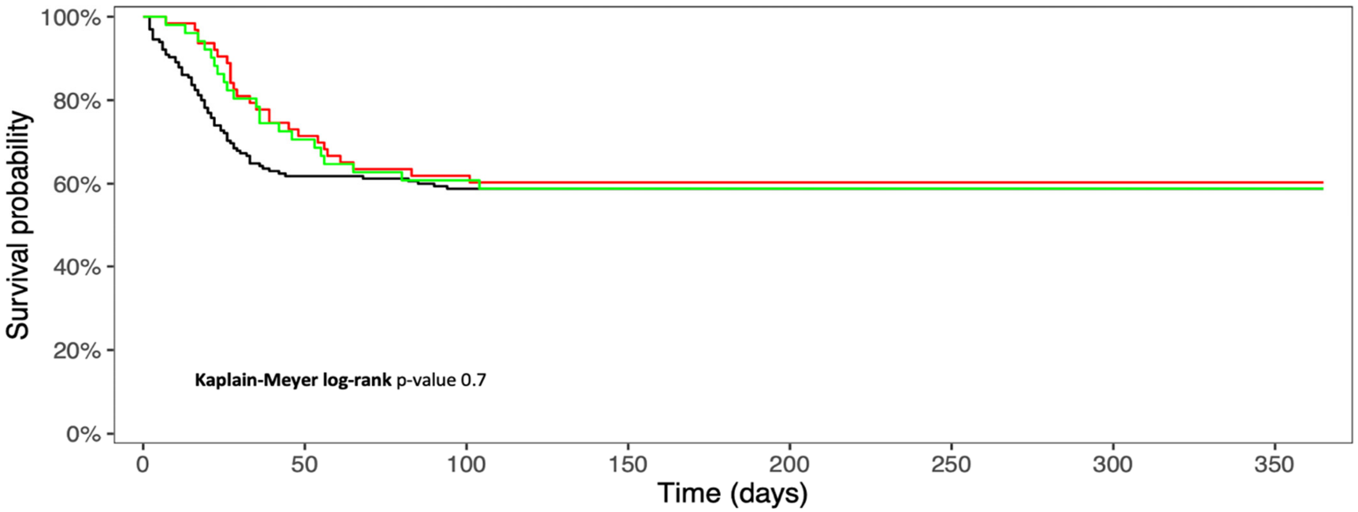

At Risk

	0	50	100	150	200	250	300	350
Absence of fungi	165	102	97	97	97	97	97	97
Fungal infections	63	45	39	38	38	38	38	38
Fungal colonizations	51	36	31	30	30	30	30	30

Events

	0	50	100	150	200	250	300	350
Absence of fungi	0	63	68	68	68	68	68	68
Fungal infections	0	18	24	25	25	25	25	25
Fungal colonizations	0	15	20	21	21	21	21	21

Additional-Figure S3. Flow-chart.

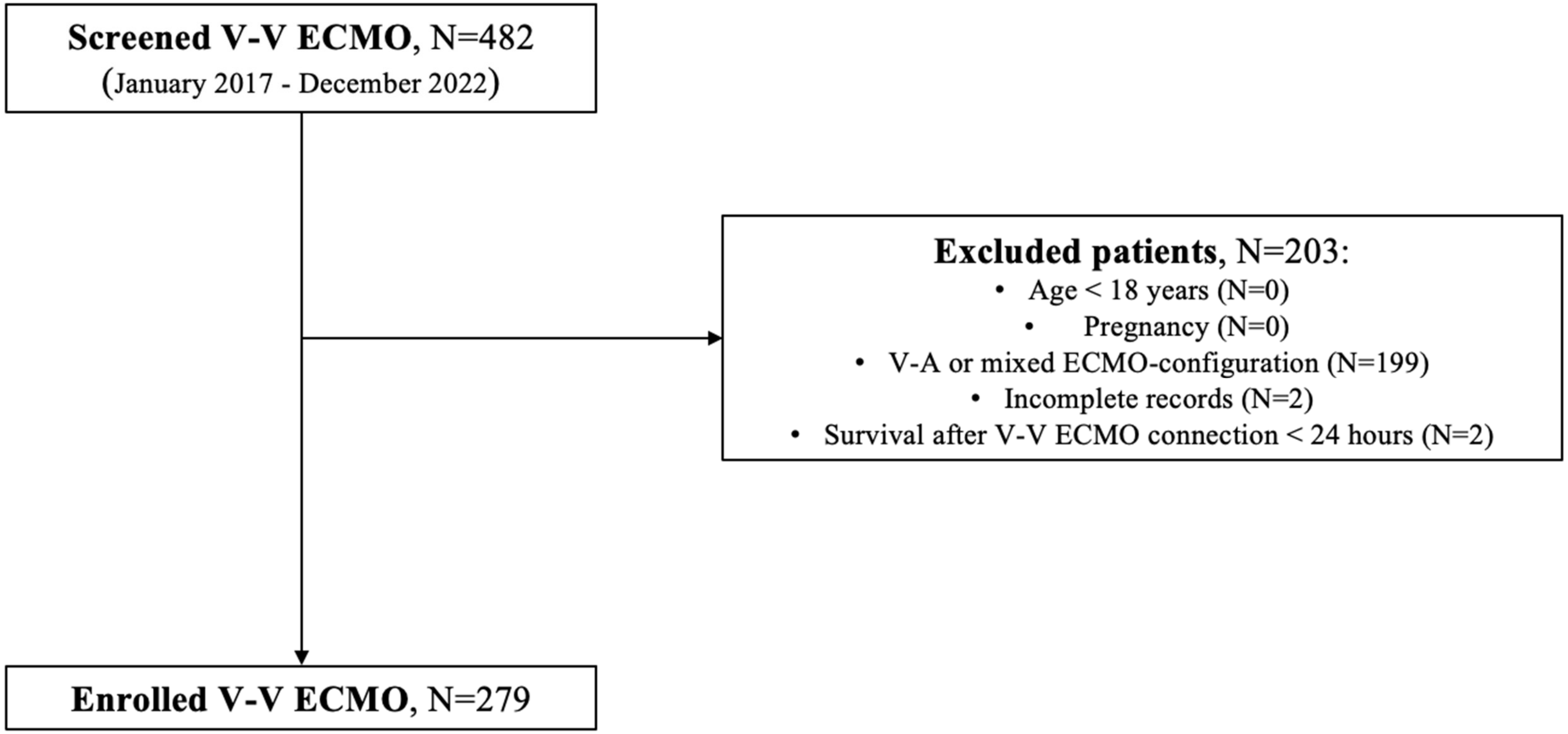

Specifically, 58 V-V ECMO patients were enrolled at Mater Domini Hospital (Catanzaro); 51 patients at Padua University Hospital; 32 at Verona University Hospital; 35 at Policlinico University Hospital (Bari); and 114 subjects at Fondazione IRCCS San Gerardo dei Tintori Hospital (Monza). *Abbreviations:* ECMO: extracorporeal membrane oxygenation; V-V: veno-venous; V-A: veno-arterial; n: number.

REEFERENCES

1. von Elm E, Altman DG, Egger M, Pocock SJ, Gøtzsche PC, Vandenbroucke JP, et al. The Strengthening the Reporting of Observational Studies in Epidemiology (STROBE) statement: guidelines for reporting observational studies. PLoS Med. 2007 Oct 16;4(10):e296.
